# Comparison of Inflammatory Marker Scoring Systems and Conventional Inflammatory Markers in Patients over 65 Years of Age Admitted to the Intensive Care Unit: A Multicenter, Retrospective, Cohort Study

**DOI:** 10.3390/jcm13144011

**Published:** 2024-07-09

**Authors:** Özlem Çakin, Arzu Karaveli, Melike Yüce Aktepe, Ayça Gümüş, Özlem Esra Yildirim

**Affiliations:** 1 Department of Internal Medicine Intensive Care Unit, Faculty of Medicine, Akdeniz University, 07070 Antalya, Türkiye; melikeyuceaktepe@akdeniz.edu.tr; 2 Department of Anesthesiology and Reanimation, University of Health Sciences, Antalya Training and Research Hospital, 07070 Antalya, Türkiye; arzukaraveli@gmail.com; 3 Department of General Medicine Intensive Care Unit, Kepez State Hospital, 07070 Antalya, Türkiye; d_r_a_y_c_a@hotmail.com; 4 Department of Internal Medicine, Faculty of Medicine, Akdeniz University, 07070 Antalya, Türkiye; creat.oey.07@gmail.com

**Keywords:** intensive care unit, inflammatory markers, scoring systems, geriatric patient

## Abstract

**Background**: The aim of the current study is to evaluate the effects of inflammation markers on infection and mortality in patients over 65 years of age monitored in the intensive care unit (ICU). In this study, we attempted to determine the significance of the pan-immune–inflammation value (PIV); the neutrophil–lymphocyte ratio (NLR); the platelet–lymphocyte ratio (PLR); the monocyte–lymphocyte ratio (MLR); the systemic immune–inflammatory index (SII); the systemic immune response index (SIRI); multi-inflammatory indices (MIIs) 1, 2, and 3; and the CRP/albumin ratio (a new biomarker) as prognostic and mortality markers in patients over 65 years of age being monitored in the ICU. **Methods**: This multicenter, retrospective, cohort study was conducted on patients aged 65 and over who were admitted to two tertiary-level ICUs. Patients with cirrhosis, bone marrow transplantation, hematologic malignancy, steroid intake, current chemotherapy treatment, and neutropenia upon admission to the ICU were excluded from this study. **Results**: A total of 333 patients were included in this study. The group’s 28-day mortality was found to be 31.8%. When each inflammatory marker associated with 28-day mortality was examined, the CRP/albumin ratio was found to be a better indicator than both the NLR and the SIRI, and the results were statistically significant (AUC: 0.665, 95% CI: 0.604–0.726, and *p* < 0.001). The NLR showed moderate discriminative ability in distinguishing mortality risk (AUC: 0.593, 95% CI: 0.526–0.660, and *p* = 0.006). Although the SIRI was lower than the NLR, it produced a statistically significant result (AUC: 0.580, 95% CI: 0.514–0.646, and *p* = 0.019). The CRP/albumin ratio was the most effective inflammatory marker in predicting mortality risk in older patients admitted to the ICU. **Conclusions**: It is important to monitor inflammatory markers (especially CRP/albumin ratio, NLR, SIRI, and MII 1-2-3) in older patients admitted to the ICU in order to accurately predict 28-day mortality. In the current study, the effects of PIV, MLR, PLR, and SII on the prediction of 28-day mortality in older ICU patients could not be demonstrated. We believe that more clinical studies are needed to determine the effects of PIV, MLR, PLR, and SII on short- and long-term prognoses and survival in older ICU patients.

## 1. Introduction

The World Health Organization (WHO), in its latest classification, categorizes individuals under 65 years old as young, those between 65 and 85 years old as young-old, and those 85 years and older as advanced-old, in parallel with the growth of the older population and their extended lifespan. Over the past few years, there has been an increasing trend in the percentage of adults aged 60 and older within the overall population [1]. Aging leads to changes in the effectiveness of the immune system, resulting in a decline in immune function with age. These changes, also known as “immune aging”, initially involve higher levels of proinflammatory cytokine secretion and a decrease in the ability of the immune system to respond to antigens. Immunological aging also leads to chronic overstimulation of the immune system and a likely increase in the risk of an irregular systemic response to infection [2].

Medical advancements in the management of acute illnesses have indeed reduced overall hospital mortality rates, but these rates remain high in older patients. Therefore, it is an urgent and crucial need in routine clinical practice to identify prognostic indicators, especially for older patients admitted to the intensive care unit (ICU) [3]. The Acute Physiologic Assessment and Chronic Health (APACHE)-II and the Sequential Organ Failure Assessment (SOFA) scoring systems are commonly used to assess the severity of acute illness and chronic outcomes in patients who are admitted to the ICU. These scoring systems, used to determine patient prognosis, also assist in resource allocation, continuous quality improvement, and the evaluation of a given treatment’s effectiveness [4]. However, recent studies have drawn attention to the importance of certain inflammatory markers obtained from the complete blood count (CBC) in determining the morbidity and mortality of individuals suffering with various diseases; this is due to the ease and cost-effectiveness of assessing such markers. New inflammatory biomarkers, such as the systemic immune–inflammatory index (SII), the systemic immune response index (SIRI), the neutrophil–lymphocyte ratio (NLR), the platelet–lymphocyte ratio (PLR), the monocyte–lymphocyte ratio (MLR), the multi-inflammatory index (MII), and the pan-immune–inflammation value (PIV), have gained popularity in assessing the mortality risk resulting from various diseases [5,6]. In a retrospective study by Yoldaş et al. [7], the effectiveness of the NLR and PLR in predicting mortality in critical patient populations was demonstrated, highlighting the ease and affordability of evaluating these markers and suggesting that high PLR and NLR in ICU patients should be closely monitored for predicting chronic outcomes. In a prospective observational study by Turan et al. [8], the effect of the PIV on predicting mortality and prognosis in patients with septic shock was evaluated; the authors demonstrated that it could effectively predict median survival time in patients with septic shock. In a study by Ayranci et al. [9] that evaluated the NLR and CRP as predictors of in-hospital mortality in geriatric emergency department patients, simultaneous elevation of the CRP and NLR was found to be a more effective predictor of in-hospital mortality, suggesting that simultaneous monitoring of the CRP and NLR by emergency department physicians may help to identify older patients at high risk of mortality at an early stage.

To the best of our knowledge, there has been no previous study evaluating the predictive value of inflammatory markers in older patients admitted to the ICU both independently of admission diagnosis and across a wide range of disease conditions and laboratory parameters. Therefore, the aim of the current study is to identify factors affecting short- and long-term prognoses and survival in patients aged 65 and over admitted to the ICU, considering the increase in the number of patients aged 65 and over and high ICU costs, and to investigate the guiding role of new inflammatory markers in patients’ survival.

## 2. Materials and Methods

### 2.1. Study Location, Duration, and Ethical Approval

The current study was conducted at the Internal Medicine Intensive Care Unit of the Akdeniz University Faculty of Medicine and the General Intensive Care Unit of Antalya Kepez State Hospital between June 2022 and December 2023. Ethical approval was obtained from the Akdeniz University Faculty of Medicine Ethics Committee prior to the study (approval number and date: KAEK-753, 28 March 2024). This study was conducted in accordance with the principles outlined in the Helsinki Declaration.

### 2.2. Study Method

This is a retrospective clinical study based on the collection of retrospective data. This research study is of an observational nature. The Strengthening the Reporting of Observational Studies in Epidemiology (STROBE) guidelines were followed for patient recording and allocation.

### 2.3. Study Population

Patients aged 65 and over who were admitted to the ICU were included in this study. Patients with missing common variable data for multivariate analysis, those followed up in the postoperative ICU, those undergoing bone marrow transplantation, those who received steroid treatment, those with cirrhosis or hematologic malignancies, those developing bone marrow suppression after chemotherapy, and those who died within the first 24 h of ICU admission were excluded from this study. For patients with multiple admissions to the ICU, only data from their first admission were considered.

### 2.4. Data Collection Methods

The necessary data for the current study were obtained from ICU patient follow-up forms, hospital electronic databases, physician daily observation notes, nurses’ observation notes, test results, and pre-ICU-admission observation forms.

### 2.5. Blood Measurements and Reference Values

Routine blood samples were taken from all patients admitted to the ICU for hemograms and biochemistry. The patients’ neutrophil, platelet, monocyte, and lymphocyte values were routinely evaluated by taking blood samples and placing them in tubes containing ethylenediaminetetraacetic acid (EDTA), which were then sent to the hospital’s laboratory center. 

Among the parameters recorded at the time of admission to the ICU and during follow-up in the ICU at the time of the secondary endpoint, the following parameters were included: platelet count (×10^9^/L; reference range: 100–300); hemoglobin (g/L; reference range: 130.0–175.0); lymphocyte count (×10^9^/L; reference range: 1.1–3.2); neutrophil count (×10^9^/L; reference range: 42.3–77.8); monocyte count (×10^9^/L; reference range: 0.4–1.2); creatinine (μmol/L; reference range: 53.0–106.0); albumin (g/L; reference range: 35.0–55.0); and C-reactive protein (CRP) (mg/L). 

### 2.6. Data Recording

The patients’ age, gender, comorbidities, lactate, neutrophil, monocyte, platelet, lymphocyte, NLR, PLR, MLR, PIV, SII, SIRI, MII 1-2-3, and CRP/albumin values, as well as their SOFA and APACHE-II scores at admission to the ICU, were routinely recorded. The values were calculated using the following formulas: PIV: [neutrophil count (10^3^/μL) × platelet count (10^3^/μL) × monocyte count (10^3^/μL)/lymphocyte count (10^3^/μL)]; NLR: [neutrophil count (10^3^/μL)/lymphocyte count (10^3^/μL)]; PLR: [platelet count (10^3^/μL)/lymphocyte count (10^3^/μL)]; SII: [neutrophil count (10^3^/μL) × platelet count (10^3^/μL)/lymphocyte count (10^3^/μL)]; MLR: [monocyte count (10^3^/μL)/lymphocyte count (10^3^/μL)]; SIRI: [neutrophil count (10^3^/μL) × monocyte count (10^3^/μL)/lymphocyte count (10^3^/μL)]; MII-1: [neutrophil count (10^3^/μL) × CRP]; MII-2: [PLR × CRP]; MII-3: [SII × CRP].

### 2.7. Statistical Analysis

The Statistical Package for the Social Sciences (SPSS) version 26 was used to statistically analyze the data obtained from the study. The variables were evaluated using Shapiro–Wilk or Kolmogorov–Smirnov tests to determine whether the variables obtained as measurement values were suitable for normal distribution. Continuous variables were expressed as the mean ± standard deviation (SD) or the median (interquartile range) according to the normality of their distribution. Categorical variables were presented as numbers (n) and percentages (%). Pearson’s chi-square test or Fisher’s exact test was used to compare categorical variables. Student’s t-test was used for parametric variables, and the Mann–Whitney U test was used for non-parametric variables. The Wilcoxon test was used to evaluate the changes between the median values of repeated (dependent) variables. Binary logistic regression analysis or linear regression analysis was used to evaluate the relationships of statistically significant variables with outcome points independently of other factors. The goodness of fit of the models was evaluated using the Hosmer–Lemeshow test. The Kaplan–Meier test, log-rank test, and Cox regression analysis were used for survival analyses. Survival curves were drawn, and differences between the groups were evaluated using Kaplan–Meier analysis. The log-rank test was used to statistically evaluate the survival differences between groups. Cox regression analysis was used to determine the factors affecting survival. In this study, a significance level of *p* < 0.05 was accepted as statistically significant.

## 3. Results

**Figure 1:**
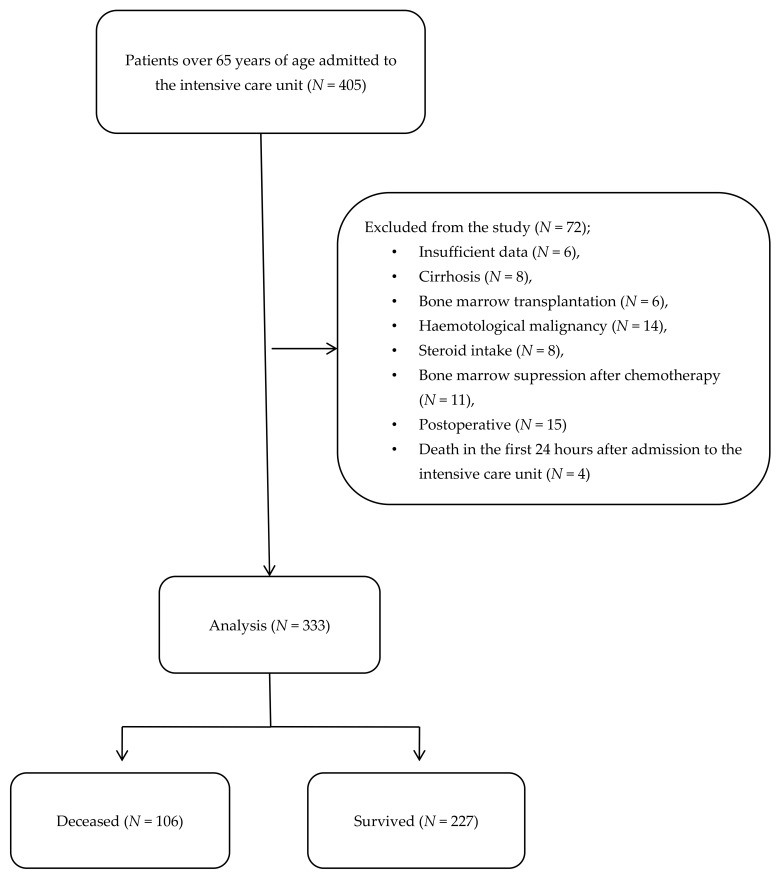
Study flow chart.

After excluding 72 patients with insufficient data, those with cirrhosis, those who had undergone bone marrow transplantation, those with a hematological malignancy, those with bone marrow suppression after recent chemotherapy, postoperative patients, and deaths in the first 24 h after admission to the ICU, a total of 333 patients were finally included. The patients were divided into two groups: the deceased group (*N* = 106) and the surviving group (*N* = 227) (Figure 1). 

Within 28 days, 31.8% were deceased, and 68.2% survived. The mean age of the deceased group was 77.2 ± 8.5 years, and it was 78.1 ± 8.6 years in the surviving group (*p* = 0.38). The distributions of sexes and chronic conditions were similar in both groups (*p* = 0.25, *p* > 0.05, respectively). The most commonly seen chronic condition was hypertension (HT), followed by diabetes mellitus (DM) (*p* = 0.40, and *p* = 0.64, respectively). The most common indication for admission to the ICU was acute respiratory failure (*p* = 0.90). This was followed by sepsis and acute kidney failure (*p* = 0.052, and *p* = 0.11, respectively). The baseline characteristics of the study population are summarized in Table 1.

The clinical characteristics of ICU patients according to 28-day mortality are presented in Table 2. Variables include culture growth, steroid use, platelet and erythrocyte transfusion, progression to sepsis in the ICU, mechanical ventilation (MV) before and in the ICU, days related to MV, hospitalization stay, ICU admission, pre- and post-ICU days, and SOFA and APACHE scores. Cultural growth rate, steroid use, and progression to sepsis were significantly higher in the deceased group (*p* < 0.001, *p* < 0.001, and *p* = 0.024, respectively). The need for ventilation before or during the ICU stay and a longer duration of ventilator use were also more common in the deceased group than in the surviving group (*p* < 0.001, *p* < 0.001, and *p* < 0.001, respectively). The length of time spent in hospital was not different between the groups (*p* = 0.68). Higher SOFA and APACHE scores were observed in the deceased group compared with the surviving group (*p* < 0.001, and *p* < 0.001, respectively).

Table 3 shows the inflammatory biomarkers and ratios related to 28-day mortality, comparing values between the deceased and the surviving group. There were statistically significant differences in the NLR, SIRI, MII1, MII2, MII3, and CRP/albumin ratios between the deceased group and the surviving group (*p* = 0.007, *p* = 0.03, *p* < 0.001, *p* < 0.001, *p* = 0.06, and *p* < 0.001, respectively). The PIV, MLR, PLR, and SII values were statistically similar between the groups (*p* = 0.47, *p* = 0.092, *p* = 0.68, and *p* = 0.22, respectively).

Each inflammatory marker related to 28-day mortality was investigated using Cox regression univariate analysis, and the results are shown in Table 4. The MLR, MII1, MII2, and MII3 did not show statistically significant associations with 28-day mortality (*p* = 0.19, *p* = 0.32, *p* = 0.37, and *p* = 0.14, respectively). Meanwhile, the NLR (HR:1.022, 95% CI: 1.006–1.038, and *p* = 0.007), SIRI (HR:1.023, 95% CI: 1.000–1.047, and *p* = 0.049), and CRP/albumin ratios (HR:1.154, 95% CI: 1.086–1.226, and *p* < 0.001) were statistically and significantly associated with 28-day mortality.

**Figure 2:**
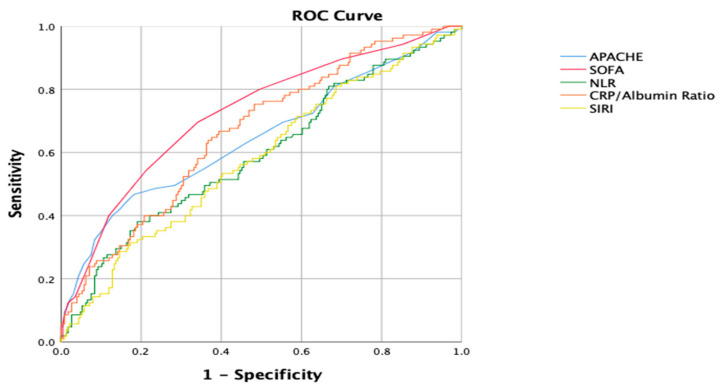
ROC curves of inflammatory markers for estimating 28-day mortality.

The predictive capability of the inflammatory markers and scoring systems for 28-day mortality risk was assessed using ROC analysis, and their cutoff values with specificity and sensitivity are presented in Figure 2 and Table 5. The NLR showed reasonable discriminatory ability in distinguishing mortality risk, with a statistically significant result (AUC: 0.593, 95% CI: 0.526–0.660, and *p* = 0.006). The SIRI also showed reasonably good discriminatory ability, though slightly lower than the NLR, with a statistically significant result (AUC: 0.580, 95% CI: 0.514–0.646, and *p* = 0.019). The CRP/albumin ratio showed moderately strong discriminatory ability and performed better than both the NLR and SIRI, with a highly statistically significant result (AUC: 0.665, 95% CI: 0.604–0.726, and *p* < 0.001). The CRP/albumin ratio was the most effective among the inflammatory markers in discriminating mortality risk in ICU patients. On the other hand, the APACHE score produced an AUC of 0.647, with a 95% CI of 0.579–0.714, and its predictive performance was statistically significant (*p* < 0.001). The SOFA score had a higher AUC of 0.723, with a 95% CI of 0.663–0.783, and was also statistically significant (*p* < 0.001). The SOFA score appeared to have the highest AUC among the predictors listed, and the CRP/albumin ratio had the highest predictive capability among the inflammatory markers.

## 4. Discussion

According to the findings of the current study, in patients aged 65 and older who were admitted to the ICU and later died, there was a higher incidence of culture positivity, steroid use, and progression to sepsis compared with patients who survived. Additionally, patients who died before or during their stay in the ICU had greater need for ventilation, and their treatment with ventilation lasted longer. Furthermore, the SOFA and APACHE-II scores were higher in deceased patients than in survivors. Regarding 28-day mortality, the NLR, SIRI, MII 1-2-3, and CRP/albumin ratios were found to be higher in deceased patients compared to survivors. However, the values of PIV, MLR, PLR, and SII did not show a significant impact on 28-day mortality. The NLR (moderate level), SIRI (moderate level), and CRP/albumin ratio (moderate level) were identified as discriminative factors in the 28-day mortality risk assessment. The CRP/albumin ratio showed better performance than both the NLR and SIRI in predicting 28-day mortality. However, despite higher SOFA scores, both the SOFA and APACHE II scores were found to have high predictive value in determining 28-day mortality in older ICU patients. 

Neutrophils and lymphocytes represent a significant percentage of all immune cells in the bloodstream, making the NLR a relatively simple inflammation index to calculate. In the current study, it was found that the NLR ratio was statistically higher in deceased patients compared to surviving patients, demonstrating the prognostic value of the NLR ratio in critical care patients aged 65 and older. It is assumed that systemic inflammation and stress lead to neutrophilia and lymphopenia, which causes the NLR ratio to increase [10]. This increased NLR—as a result of an increase in the neutrophil count and secondary to an increase in the granulocyte series during inflammation, alongside a decrease in the lymphocyte count during inflammatory stress—may serve as an indicator of systemic inflammation [7]. Additionally, increases in the NLR have been attributed to increased neutrophil production and lymphocyte apoptosis during inflammatory stress [11]. In a prospective cohort study involving 5034 patients that aimed to predict mortality in older patients admitted to the hospital, in accordance with our study’s findings, deceased patients were found to have significantly higher NLR ratios than survivors during their hospital stay. The study also suggested that the NLR could facilitate the early identification of older patients at high risk of death because it is easily applicable and cost-effective [3]. In another study evaluating the effect of the NLR on postoperative ICU admission and mortality in older patients undergoing hip surgery, a high NLR value was found to be a strong predictor of admission to the ICU and subsequent mortality in older patients [12]. 

SIRI, a novel prognostic marker based on the counts of neutrophils, monocytes, and lymphocytes, reflects the balance between the inflammatory response and immune status [13]. It was first identified in a study involving patients with hepatocellular carcinoma (HCC) in 2014. It has been described as a strong prognostic indicator of poor outcomes and a promising marker for directing treatment strategy in HCC [14]. Subsequently, it has been reported that SIRI elevation could be used in the diagnosis of certain cancers, particularly gastric cancer [15,16] and colorectal carcinoma [17], indicating worsening inflammatory processes and a poor prognosis in these patients. In a study evaluating the relationship between inflammatory mechanisms and intracerebral hemorrhage in pneumonia, although the NLR showed a stronger association with the of severity pneumonia, the SIRI exhibited high predictive accuracy in the early recognition of pneumonia severity after intracranial hemorrhage as well as an association with poor neurological outcomes upon discharge [18]. However, in the literature, data evaluating the predictive value of the SIRI for mortality and prognoses in elderly patients admitted to the ICU are scarce. In the present study, we found that although the SIRI was less effective than the CRP/albumin ratio in predicting 28-day mortality in critically ill older patients, it can still function as a strong indicator, making ours the first study to demonstrate the independent significance of the SIRI as a predictor of mortality in critically ill older patients.

In our study, the CRP/albumin ratio was identified as an independent risk factor for 28-day mortality in critical patients, and our findings are consistent with those of previous studies. Despite the numerous scoring systems used to predict the prognosis of critical patients, the CRP/albumin ratio remains valuable. This is because it is a relatively simple marker that is easy to use in all settings. Produced in response to various cytokines after infection, ischemia, trauma and other inflammatory conditions, CRP is an acute-phase protein [19]. Many studies [20,21] have demonstrated an association between high CRP levels and low serum albumin levels and prognosis and mortality in critical patients. Additionally, it has been noted in the literature that the CRP/albumin ratio is more consistent in predicting prognosis when compared with CRP or albumin alone [22]. In our study, we found the CRP/albumin ratio to be a biomarker with high value in predicting the survival of older patients; data to support this statement are available in the literature. The CRP/albumin ratio is an independent determinant of 28-day mortality in ICU patients, and a higher CRP/albumin ratio is associated with increased mortality in critical patients, according to a retrospective study by Par et al. [23]. A high CRP/albumin ratio upon admission to the emergency department is an independent determinant of all-cause in-hospital mortality in patients aged 65 and older, according to another study evaluating the CRP/albumin ratio as a determinant of in-hospital mortality in geriatric patients presenting to the emergency department [24].

MIIs were initially developed by Gardini et al. [25]. The ongoing Italian Trial in Advanced Colorectal Cancer (ITACa) study has demonstrated the usefulness of all MIIs as prognostic markers in patients with metastatic colon cancer. The MIIs allow for the evaluation of hematological parameters—such as neutrophils, platelets, and lymphocytes—along with the chronic inflammatory marker CRP, thereby acting as powerful markers of systemic conditions [26]. They are deemed strong, easy-to-use, and practical markers for the early prediction of mortality in COVID-19 patients at high risk of mortality in the ICU [27]. Studies in the literature have also indicated that the MIIs could function as an independent determinant in ischemic stroke patients [28]. The results of the current study represent the first demonstration of the independent significance of all MIIs as determinants of mortality in critically ill geriatric patients. However, given the limitations of the data in the literature, we believe that more studies are needed to evaluate the prognostic importance of both SIRI and all MIIs in critical care geriatric patients.

In our study, no relationship was observed between PIV, NLR, PLR, and SII values and mortality. Various studies concerning these markers can be found in the literature. Recent studies have shown that the PIV can serve as an inflammatory marker for various diseases. In the literature, high PIV levels have been found to correlate with poor prognoses in cancer patients [6]. A prospective study conducted on 82 septic patients in the ICU investigated the relationship between the PIV and sepsis; however, no statistically significant relationship was found between mortality and the PIV [8]. In a study by Mangalesh et al. [29], the NLR, PLR, and SII were shown to be independent determinants of in-hospital mortality in septic patients, with SII performing better than both the NLR and PLR; moreover, the addition of the SII to the SOFA score led to a significant improvement in prognostic performance. A total of 5537 patients were analyzed in a study wherein the data of septic patients were examined, and a significant relationship was found between the PLR and mortality only in subgroups without vasopressor use and without AKI and those with a SOFA score of ≤10 [30]. In a study evaluating decreased cognitive abilities in older patients after surgery, an increased SII was found to be an independently associated risk factor for decreased cognitive abilities after surgery [31].

### Limitation

The current study has several limitations. First, the laboratory data used in the study were collected on the first day of admission to the ICU; therefore, continuous changes could not be analyzed. Second, this was a retrospective observational study, and selection bias and confounding bias are inevitable in studies of this kind. Finally, the current study was multicenter; however, a small number of patients were involved, meaning the results may not necessarily be representative of larger populations. Future larger, multicenter prospective studies are needed to confirm or refute the results of this study.

## 5. Conclusions

In geriatric patients admitted to the ICU, it has been determined that (among the scoring systems used in the ICU) the SOFA score and (among the inflammatory markers) the CRP/albumin ratio have the greatest ability to predict 28-day mortality. Monitoring of the NLR, SIRI, MII 1-2-3, and CRP/albumin ratios is recommended for elderly ICU patients. Nevertheless, the effects of PIV, MLR, PLR, and SII values on predicting 28-day mortality could not be demonstrated in the current study. Therefore, we believe that further clinical studies are needed to determine the effects of the PIV, MLR, PLR, and SII on short- and long-term prognoses and survival in geriatric patients admitted to the ICU.

## Figures and Tables

**Table 1:** Baseline characteristics of patients according to in-hospital mortality and 28-day mortality (values presented as mean ± standard deviation or number (percentage)).

	28-Day Mortality		
	Deceased (*N* = 106)	Surviving (*N* = 227)	*p*-Value
Age, years	77.2 ± 8.5	78.1 ± 8.6	0.38
Sex, female	47 (44.3)	116 (51.1)	0.25
Chronic Diseases			
Diabetes mellitus	43 (40.6)	86 (37.9)	0.64
Hypertension	48 (45.3)	114 (50.2)	0.40
Hyperlipidemia	5 (4.7)	7 (3.1)	0.46
Coronary artery disease	12 (11.3)	31 (13.7)	0.55
Atrial fibrillation	15 (14.2)	24 (10.6)	0.34
Chronic kidney disease	13 (12.3)	22 (9.7)	0.48
Pulmonary disease	19 (17.9)	46 (20.3)	0.62
Cerebrovascular disease	10 (9.4)	37 (16.3)	0.94
Multimorbidity	72 (67.9)	131 (57.7)	0.075
Diagnosis in ICU			
Sepsis	37 (34.9)	56 (24.7)	0.052
Acute respiratory failure	47 (44.3)	99 (43.6)	0.90
Acute kidney disease	13 (12.3)	16 (7.0)	0.11
Pancreatitis	0	3 (1.3)	0.55
Trauma	1 (0.9)	7 (3.1)	0.24
Hepatorenal syndrome	1 (0.9)	2 (0.9)	0.95
Cardiorenal syndrome	1 (0.9)	2 (0.9)	0.95
Cerebrovascular event	6 (5.7)	19 (8.4)	0.38
Hemorrhagic shock	7(6.7)	20 (8.8)	0.50
Postoperative care	9 (8.5)	26 (11.5)	0.41

ICU: intensive care unit.

**Table 2:** Clinical characteristics of patients in the intensive care unit according to 28-day mortality (values presented as median (interquartile range) or number (percentage)).

	Deceased (*N* = 106)	Surviving (*N* = 227)	*p*-Value
Culture growth	71 (67.0)	93 (41.0)	<0.001
Steroid use	71 (67.0)	87 (38.3)	<0.001
Platelet tx	13 (12.3)	15 (6.6)	0.083
Erythrocyte tx	48 (45.3)	86 (37.9)	0.20
Sepsis in ICU	64 (60.4)	107 (47.1)	0.024
MV before ICU	26 (24.5)	14 (6.2)	<0.001
MV in ICU	64 (60.4)	16 (7.0)	<0.001
Day of MV	3.0 (3.0)	0.0 (2.0)	<0.001
Day of hospitalization	13.5 (18.3)	12.0 (12.0)	0.68
Day of ICU	7.0 (10.0)	6.0 (5.0)	0.016
Day before ICU	1.5 (11.0)	0.0 (1.0)	<0.001
Day after ICU	0.0 (0.0)	4.0 (7.0)	<0.001
SOFA score	6.0 (4.0)	3.0 (3.0)	<0.001
APACHE-II score	24.5 (15.3)	20.0 (8.0)	<0.001

APACHE: Acute Physiologic Assessment and Chronic Health; ICU: intensive care unit; MV: mechanical ventilation; SOFA: Sequential Organ Failure Assessment; tx: transfusion.

**Table 3:** Inflammatory markers of patients according to 28-day mortality (values presented as median (interquartile range)).

	28-Day Mortality		
	Deceased (*N* = 106)	Survived (*N* = 227)	*p*-Value
PIV	1104.3 (2003.4)	952.0 (1969.8)	0.47
NLR	11.6 (20.2)	9.3 (11.6)	0.007
MLR	0.6 (0.6)	0.5 (0.6)	0.092
SIRI	5.8 (11.2)	4.5 (8.3)	0.03
PLR	223.2 (258.8)	203.5 (240.7)	0.68
SII	22.2 (26.6)	26.7 (29.3)	0.22
MII1	813.8 (2153.1)	416.3 (1997.1)	<0.001
MII2	20.182 (46.303)	6993.6 (28.048.2)	<0.001
MII3	2026.9(4630.6)	965.5 (3134.3)	0.006
CRP/Albumin	4.03 (6.5)	1.6 (4.7)	<0.001

CRP: C-reactive protein; MII: multi-inflammatory index; MLR: monocyte–lymphocyte ratio; NLR: neutrophil–lymphocyte ratio; PIV: pan-immune–inflammation value; PLR: platelet–lymphocyte ratio; SII: systemic immune–inflammatory index; SIRI: systemic immune response index.

**Table 4:** Cox regression analysis of inflammatory markers of patients as they relate to 28-day mortality (univariate analysis, entry method).

	Hazard Ratio	95% CI	*p*-Value
NLR	1.022	1.006 to 1.038	0.007
MLR	1.267	0.891 to 1.801	0.19
SIRI	1.023	1.000 to 1.047	0.049
MII1	1.0	1.0 to 1.0	0.32
MII2	1.0	1.0 to 1.0	0.37
MII3	1.0	1.0 to 1.0	0.14
CRP/Albumin	1.154	1.086 to 1.226	<0.001

CRP: C-reactive protein; MII: multi-inflammatory index; MLR: monocyte–lymphocyte ratio; NLR: neutrophil– lymphocyte ratio; SIRI: systemic immune response index.

**Table 5:** ROC curve analysis of the possible predictors of 28-day mortality.

	Cut-Off	Sensitivity	Specificity	AUC	95% CI	*p*-Value
APACHE	21.5	59.0	58.8	0.647	0.579 to 0.714	<0.001
SOFA	4.5	69.5	65.9	0.723	0.663 to 0.783	<0.001
NLR	7.79	61.9	46.9	0.593	0.526 to 0.660	0.006
SIRI	5.00	56.2	54.9	0.580	0.514 to 0.646	0.019
CRP/Albumin	2.15	70.5	55.3	0.665	0.604 to 0.726	<0.001

APACHE: Acute Physiologic Assessment and Chronic Health; CRP: C-reactive protein; NLR: neutrophil–lymphocyte ratio; SIRI: systemic immune response index; SOFA: Sequential Organ Failure Assessment.

## Data Availability

The data presented in this study are available upon request from the corresponding author. The data have not been made publicly available in order to maintain participants’ privacy.
